# Boolean approach to signalling pathway modelling in HGF-induced keratinocyte migration

**DOI:** 10.1093/bioinformatics/bts410

**Published:** 2012-09-03

**Authors:** Amit Singh, Juliana M. Nascimento, Silke Kowar, Hauke Busch, Melanie Boerries

**Affiliations:** ^1^Freiburg Institute for Advanced Studies, LifeNet, Albert-Ludwigs-University of Freiburg, Albertstrasse 19; ^2^Center for Biological Systems Analysis, Albert-Ludwigs-University of Freiburg, Habsburger Strasse 49, 79104 Freiburg, Germany

## Abstract

**Motivation:** Cell migration is a complex process that is controlled through the time-sequential feedback regulation of protein signalling and gene regulation. Based on prior knowledge and own experimental data, we developed a large-scale dynamic network describing the onset and maintenance of hepatocyte growth factor-induced migration of primary human keratinocytes. We applied Boolean logic to capture the qualitative behaviour as well as short-and long-term dynamics of the complex signalling network involved in this process, comprising protein signalling, gene regulation and autocrine feedback.

**Results:** A Boolean model has been compiled from time-resolved transcriptome data and literature mining, incorporating the main pathways involved in migration from initial stimulation to phenotype progress. Steady-state analysis under different inhibition and stimulation conditions of known key molecules reproduces existing data and predicts novel interactions based on our own experiments. Model simulations highlight for the first time the necessity of a temporal sequence of initial, transient MET receptor (met proto-oncogene, hepatocyte growth factor receptor) and subsequent, continuous epidermal growth factor/integrin signalling to trigger and sustain migration by autocrine signalling that is integrated through the Focal adhesion kinase protein. We predicted *in silico* and verified *in vitro* that long-term cell migration is stopped if any of the two feedback loops are inhibited.

**Availability:** The network file for analysis with the R BoolNet library is available in the Supplementary Information.

**Contact:** melanie.boerries@frias.uni-freiburg.de or hauke.busch@frias.uni-freiburg.de

**Supplementary information:** Supplementary data are available at *Bioinformatics* online.

## 1 INTRODUCTION

Cell migration and wound healing are complex cellular processes that involve keratinocytes, fibroblasts, blood vessels and inflammatory cells (Xue *et al.*, 2007). Keratinocyte migration plays an important role in re-epithelialization and wound healing (Hunt *et al.*, 2000), which is a multistep cellular process by the coordination of extra- and intracellular signals (Muyderman *et al.*, 2001; Werner *et al.*, 2007). The precise regulation of cell migration in its temporal sequence, activation and de-activation is crucial for tissue homeostasis. In its aberrant form, it can lead to scar formation (Heng, 2011) and has critical implications to cancer metastasis formation (Schäfer and Werner, 2008).

Different growth factors such as hepatocyte growth factor (HGF), epidermal growth factor (EGF), transforming growth factor-beta (TGF-*β*), keratinocyte growth factor (KGF) and fibroblast growth factor (FGF) that activate and regulate cell migration have been extensively studied in many cell types (Birchmeier *et al.*, 2003; Hudson and McCawley, 1998; Jaakkola *et al.*, 1998; Pastore *et al.*, 2008; Tsuboi *et al.*, 1993). These growth factors have been found to overlap with mitogen-activated protein kinase (MAPK) pathways (Cho and Klemke, 2000; Kain and Klemke, 2001; Klemke *et al.*, 1997).

HGF interacts and activates MET receptor (Bottaro *et al.*, 1991) to induce context-dependent several cellular processes such as proliferation, cell movement or morphogenic differentiation (Brinkmann *et al.*, 1995; Clague, 2011; Jeffers *et al.*, 1996; Medico *et al.*, 1996). Herein, we focus on HGF-induced migration of primary normal human keratinocytes (NHK).

Although there is vast literature concerning HGF-induced keratinocyte migration and MET receptor dynamics, the dynamic interplay of initial MET receptor regulation and subsequent autocrine regulation that initiate, sustain and control cell migration remain poorly understood. Based on time-resolved transcriptome data of NHK after HGF stimulation, we have previously inferred a gene regulatory model describing the decision process of NHK cells towards migration (Busch *et al.*, 2008). From the model analysis it was evident that several pathways coordinate their action to initiate and sustain cell migration upon initial HGF stimulation: migration is started through the AP-1 system and maintained after MET receptor internalization (Clague, 2011) by autocrine signalling through EGF receptor (EGFR) and urokinase plasminogen activator surface receptor (uPAR) (Schnickmann *et al.*, 2009). The model predicted qualitatively how the temporal sequence of transient MET receptor activation and subsequent long-term EGF receptor activity sustained the migratory phenotype. However, as the model was based on transcriptome data alone, there was no mechanistic explanation of the observed processes. A model combining transcriptome data with mechanistic protein signalling has been missing so far. A major obstacle in building such a model lies in the different time scales involved in the process of cell migration. In general, the transcriptome response changes over several hours, while protein signalling pathways become active within minutes upon receptor stimulation (Mesecke *et al.*, 2011). Capturing all necessary and sufficient events on the protein signalling level, including kinetic parameters, is close to impossible by current biological technology.

To link our prior transcriptome-based model with protein signalling pathways, we present a Boolean network model of HGF-induced keratinocyte migration. The Boolean approach allows to derive important functional properties and predictions without the need for detailed quantitative kinetic data and parameters. In the past, the approach has been successfully applied for diverse systems such as gene regulatory networks (Albert and Othmer, 2003; Chaves *et al.*, 2005), models of floral morphogenesis (Mendoza *et al.*, 1999), mammalian cell cycle (Mendoza, 2006), EGFR signalling (Samaga *et al.*, 2009) or apoptosis (Schlatter *et al.*, 2009).

To our knowledge, this is the first model for HGF-induced keratinocyte migration that incorporates protein signalling, gene regulation and autocrine feedback, following cellular dynamics from initial stimulation to the execution of the phenotype. To obtain the dynamical behaviour reproducing literature knowledge and our own experimental data, we include several time scales in the model mimicking the fast activation of downstream signalling of MET, MAPK/ERK and p38/JNK pathways, as well as the slow transcriptome response and subsequent autocrine activation of EGFR and uPA receptors, all of which are necessary to sustain cell migration after MET receptor internalization. Specifically, from a logical steady-state analysis, we show that priming of the HGF–MET receptor system is necessary for continued autocrine regulation through EGFR and integrins, sustaining the MAPK/ERK activity. More importantly, we predicted and showed experimentally, that the inhibition of the plasminogen activator inhibitor-1 (PAI-1), or serpin E1, a serine protease inhibitor, stops cell migration only beyond 1 h of stimulation, when autocrine signalling loops through uPA/uPAR become important and after the first wave of protein signalling and transcriptional response.

## 2 METHODS

### 2.1 Reconstruction of the NHK migration model

A Boolean network, comprising protein signalling pathway, gene expression dynamics and autocrine feedback was constructed based on our previous gene regulatory model for keratinocyte migration (Busch *et al.*, 2008). There, time-resolved gene expression data of NHK were recorded at *t* = [0h, 1h, 2h, 3h, 4h, 6h, 8h] after stimulation with HGF (ArrayExpress ID: E-TABM-440). As a basis for our HGF-induced cell migration network, we chose genes that have either a large differential response after HGF stimulation or genes that are known to be functionally related to cell migration. Genes finally included in the model are depicted in Figure 2A. To link immediate early and late responding genes to initial MET receptor signalling and subsequent protein pathways, respectively, we integrated differentially expressed genes with signalling pathways known from literature. Additional pathways for the HGF migration network were identified through the commercial IPA (Ingenuity Systems; www.ingenuity.com) software. A dataset containing gene identifiers and corresponding expression values was uploaded into the application. Each gene kinetic was mapped to its corresponding object in the Ingenuity Knowledge Base. The above identified molecules were overlaid onto a global molecular network developed from information contained in the application. This generated a score with meaningful and significant networks, biological functions and the canonical pathways based on the Fisher exact test.

### 2.2 The NHK migration model as a dynamic Boolean model

We used a Boolean model framework to construct a dynamic, temporally discrete model for NHK migration. Each node *y* can take the values 0 or 1, representing either the present/absent or inactive/active, i.e. phosphorylated, state of the protein or gene. The network state is represented by the vector with the set of Boolean variables *Y* = {*y*_1_,*y*_2_,...,*y_n_*}, where *y_i_* denotes the state of the *i*th node. The state of activation of each node changes according to the transition function *F* = {*f*_1_,*f*_2_,...,*f_n_*}. The next state of the network *Y* (*t* + 1) changes in discrete time steps according to *y_i_*(*t* + 1) = *f_i_*{**x**(*t*)}.

We simulated the Boolean network under synchronous update using the R BoolNet library (Müssel *et al.*, 2010). As we consider both protein signalling and gene regulation, two time scales were included in the model. Rapid protein modifications such as phosphorylation can thereby be separated from long-term effects, transcriptional changes, protein synthesis and autocrine signalling. Time-scale separation was done by first introducing a reference time through the transcriptome kinetics and equating the time of maximal fold change of the respective genes with their switching-on time. Consequently, our model contains two time scales: 0–1 and 1–3 h (marked as 1 and 3, respectively), denoting the time intervals after HGF stimulation, during which the reactions can be switched on the earliest. These represent the early HGF downstream signalling and first transcriptional response as well as the autocrine feedback, which are both necessary to trigger and sustain cell migration.

The reference publications from which the interactions have been inferred as well as their Boolean transition functions and time windows are listed in the Supplementary Table S1.

### 2.3 Cell culture

Normal human skin keratinocytes (NHK) were derived from foreskin epidermis and cultivated in keratinocyte serum free medium (KSFM; Invitrogen, Carlsbad, CA, USA) as previously described (Busch *et al.*, 2008). Cells were kept under a humidified environment with 5% CO_2_ and 37°C. NHK up to Passages 4–5 were used in all experiments of this study. Cells were treated as described bellow and collected after 1, 2, 3, 4, 6 and 8 h treatment for expression profiling.

### 2.4 Scratch assay

Monolayer scratch assays were used to evaluate migration of NHK as described before (Busch *et al.*, 2008). Briefly, cells were grown to confluence in ibidi *μ*-dish containing culture inserts (ibidi, Munich, Germany) and treated with mitomycin c 10 *μ*g/mL (Sigma-Aldrich, St. Louis, MO, USA) for 3 h before stimulation. Cells were stimulated with 10 ng/mL HGF (Sigma-Aldrich) and/or 25 *μ*M Tiplaxtinin, a PAI-1 inhibitor (Axon Medchem, Groningen, Netherlands). Time-lapse microscopy of cell migration was recorded using the Perfect Focus Systems, every 30 min up to 24 h, on a Nikon Eclipse Ti microscope with a Digital Sight DS-QiMc (Nikon Instruments Inc., Tokyo, Japan), coupled to an ibidi-heating chamber.

### 2.5 Cell migration assay

Keratinocyte migration was analysed with an independent second technique, so-called xCELLigence Real-Time Cell Analyzer DP (Roche Diagnostics). The specific migration CIM-plates were filled with medium and the respective stimulus/inhibitor as described above. NHK were seeded (6 × 10^4^ cells/well) into the top chamber wells of the CIM-plate according to the manufacturer instructions. Cell migration was monitored every 15 min for up to 24 h by changes of the impedance signal of the cells that crossed the membrane from the top to the bottom chamber. For analysis of migration, the area under the curve was measured for the first 8 h. The data were expressed as the mean ± SD of quadruplicates in three independent experiments. Differences were assessed by the Student *t*-test for unpaired samples and a *P*-value < 0.05 was considered to be significant.

### 2.6 Western blot

NHK were lysed in RIPA buffer containing protease inhibitor cocktail (Roche), and later diluted in Laemmli buffer. Proteins were electrophoresed on 12.5% sodium dodecyl sulphate (SDS)-polyacrylamide gels, transferred to polyvinylidene difluoride membranes, and immunoblotted with antibodies to total p44/42 MAPK (ERK1/2) (Cell Signalling #9102), phospho p44/42 MAPK (pERK1/2) (Cell Signalling #9101), total FAK (Cell Signalling #3285), phospho-FAK (Tyr925) (Cell Signalling #3284) overnight at 4°C. Membranes were visualized with chemiluminescence after using the appropriate horseradish peroxidase-linked secondary antibody (Sigma-Aldrich). Immunoblots were quantified using Multi Gauge v3.1 (Fujifilm) software. Values obtained for both p44 and p42 ERK bands were added together and phospho p44/42 values were normalized to total p44/42. Values of phospho-FAK (Tyr925) were normalized to total FAK.

## 3 RESULTS AND DISCUSSION

Upon stimulation with HGF keratinocytes start to migrate collectively. Several points of interference that modulate keratinocyte migration have been previously identified. However, time sequential orchestration of the whole-cell signalling remains unclear so far. Our goal is to understand how downstream signalling of the HGF-activated MET receptor is translated into a sustained migratory behaviour. To accomplish this, we developed a Boolean network model of the combined MAPK signalling pathways, gene regulation and autocrine feedback, which links known interactions of downstream protein and gene targets of MET with subsequent changes in the cellular homeostasis.

### 3.1 Boolean network model properties

**Fig. 1.**
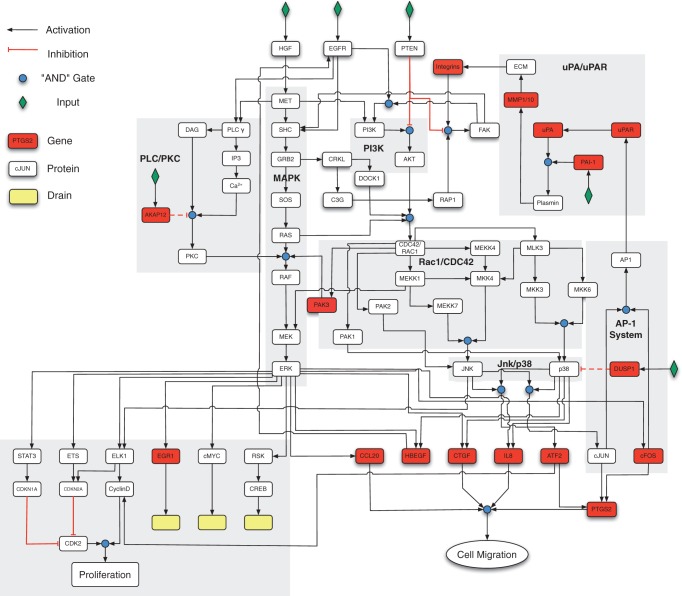
Boolean network model of the HGF-induced keratinocyte migration. Nodes are connected by directed edges, where black and red connections denote activating and inhibitory interactions, respectively. Red nodes represent transcriptionally regulated proteins, yellow nodes are endpoints of the network. ‘AND’ ‘gates are denoted by blue dots and ‘OR’ gates are found where more than one edge connects to single node. Dashed edges denote interactions that have not been considered, when calculating the steady state and are shown for completeness

The keratinocyte migration model is a logical interaction hypergraph connected by logic gates. It comprises 66 nodes, excluding the drain nodes and 66 interactions (Fig. 1), integrating the main pathways and genes known to be involved in HGF-induced keratinocyte migration. For detailed information about the biological processes and context from which the model was derived, please confer to Supplementary Table S1.

**Fig. 2.**
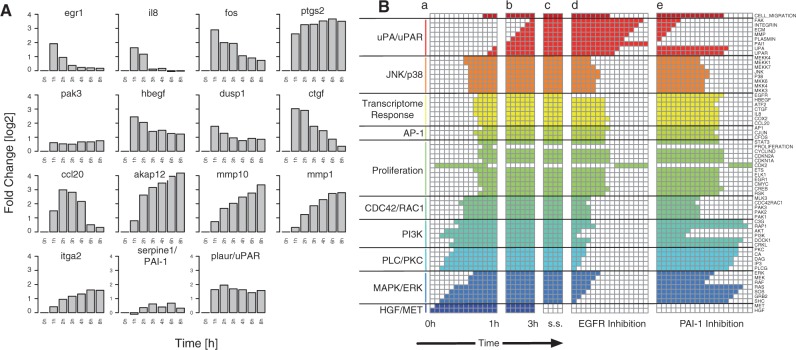
(**A**) Transcriptome time series of differential regulation with respect to 0 h time point for genes included in the Boolean cell migration network. (**B**) Network simulation of time sequential pathway activation. (a) Path to attractor upon HGF stimulation up to 1 h after stimulation. (b) Change in network state after autocrine signalling through uPA and integrin signalling up to 3 h after stimulation, initializing the network using the network state after 1 h and setting PAI-1 activity to 1. (c) Switching off MET receptor signalling after 3 h. Cell migration sustains in steady state (s.s.). (d) and (e) Inhibition of EGFR and PAI-1, respectively, in both cases leading to a stop in cell migration. Time increases in arbitrary units from left to right until a logical steady state is reached. Absolute times correspond to the maximal fold induction of the corresponding transcriptome data. Pathway-based grouping of network nodes (cf. Fig. 1) is indicated in colour and named on the left

There are two input nodes, the MET receptor, stimulated through HGF, as well as EGFR, stimulated by HBEGF. Both receptors have been shown to be involved in keratinocyte migration in a time-sequential manner (Busch *et al.*, 2008). Four further nodes are included to applying external interventions: PAI-1, AKAP12, DUSP1 and PTEN. AKAP12 and DUSP1 are not included in the calculation of the steady-state below. Despite the fact that they are found to be up-regulated on the transcriptome level, the respective proteins seem to exert a stabilizing negative feedback function (Legewie *et al.*, 2008), not completely inhibiting their specific targets, but allowing for a rapid protein induction (Blüthgen, 2010). In line with this, we find *akap12* strongly up-regulated in HGF-induced keratinocyte migration (Fig. 2A), although over-expression of *akap12* has been associated with reduced motility (Gelman, 2010). ABoolean representation of such negative feedback behaviour is not straight forward and will often lead to — a biologically questionable — oscillatory steady state. Hence, we included the proteins for completeness, yet excluded it from steady-state analysis.

### 3.2 Dynamics of the Boolean network model for migration

To search for steady-state attractors of the Boolean network model, we randomly initialized all network nodes, except for HGF/PAI-1 and AKAP12/PTEN with either 0 or 1 and performed synchronous state transitions until a simple attractor was reached. Activating the nodes HGF and PAI-1 to 1 mimicked HGF stimulation and allowed for autocrine feedback through uPAR signalling. Setting AKAP12 and PTEN to zero excluded their inhibitory effects. Sampling over *n* = 10^7^ random initialization of the network we find only one attractor of the network with the node CELL MIGRATION switched on. Although only a minor fraction of all possible network initializations (*n* = 2^62^) has been sampled, this result is still suggestive of a robust response irrespective of the initial network state, i.e. cell migration follows upon MET receptor activation and subsequent autocrine signalling.

In detail node and pathway activity moves along the following steps after transient MET receptor signalling: (i) the first and necessary input into the network towards migration is the stimulation with HGF, which immediately and specifically activates the MET receptor. Within the first hour, the signal activates three different downstream pathways, PLC/PKC, MAPK/ERK and PI3K (grey boxes, Fig. 1); (ii) ERK phosphorylation activates transcriptional responses, leading to the down-regulation of proliferation and activates, together with p38/JNK, essential cytokines and transcription factors for migration within the first hour, such as HBEGF, IL8 and ATF2, as well as cJUN and cFOS, respectively. HBEGF has been shown to mediate the subsequent autocrine activation of EGFR (Busch *et al.*, 2008); (iii) The activation of cJUN and cFOS nodes leads to an initiation of the AP-1 system, which in turn stimulates the uPA/uPAR signalling pathway by activation of the uPAR, triggering the formation from plasminogen activator to plasmin. According to the transcriptome kinetics, the uPA/uPAR pathway becomes active 2 h after HGF stimulation, being controlled by PAI-1. Plasmin is a major factor for induction of metalloproteinases 1 and 10 (MMP1/MMP10), linking degradation of the extracellular matrix (ECM) with integrin signalling; (iv) The integrins transmit the extracellular signalling back into the cell through the focal adhesion kinase (FAK). Together with activated EGFR this protein sustains PLC/PKC, MAPK and PI3K activity similar to the initial HGF/MET activation. It is known that the MET receptor undergoes rapid internalization, possibly switching off its signalling in favour of EGFR activity. In fact, we have shown previously that HGF/MET activity is not required for keratinocyte migration beyond 1.5 h after stimulation (Busch *et al.*, 2008). Accordingly, the model suggests that continued PI3K up-regulation can only occur through the combined activity of integrin and EGFR signalling in the presence of FAK. Signalling continues through AKT, DOCK1 and RAS to sustain RAC1/CDC42 activity, which result in the downstream activity of p21 protein (CDC42/RAC)-activated kinases (PAKs), mitogen-activated kinase kinases (MKK) and finally activation of JNK/p38. This closes the autocrine loop through time sequentially activated external receptors. This late response of the keratinocyte migration network through activation of uPAR, integrins and EGFR triggers similar pathways as the MET signalling, but furthermore results in the prolonged activation of PLC/PKC, MAPK/ERK, PI3K, RAC1/CDC42 and JNK/p38 pathways to sustain the long-term migration response.

Figure 2B shows a simulation of the network dynamics after HGF stimulation up to 3 h, as marked by the white vertical gaps, having initialized all network nodes, except for HGF with 0 for clarity. The transitions towards the final steady states are indicated in arbitrary time units. Clearly, an activation wave of the three downstream pathways PLC/PKC, MAPK/ERK and PI3K is evident, resulting in the up-regulation of the respective target genes (Fig. 2A), the AP-1 system and starting cell migration within 1 h. In the following 2 h, autocrine signalling loops activate EGFR and integrin signalling through HBEGF and uPAR, respectively. Switching off the MET receptor has no influence on the steady state at this time, and cell migration continues. Switching off either uPA/uPAR signalling through PAI-1 or inhibiting EGFR stops migration through subsequent switching off all pathways.

Table 1 lists the steady states of the Boolean network under different input settings and/or different scenarios. Interestingly, the model predicts that cells will migrate only when HGF signalling primes the cells, followed by EGFR and integrin signalling. This is in line with our previous findings, which now can be explained on the causal level of protein signalling.

**Table 1.** Predicted network steady states under different network perturbations

HGF	EGF	Inhibition	Over-expression	Cell migration	Confirmed
1	0	—	—	1	Own data
0	1	—	—	0	Predicted
0	1	FAK	—	0	Predicted
1	1	EGFR	—	0	Own data
1	0	uPAR	—	0	19020551
1	0	PAI-1	—	0	Own data
1	0	PTGS2	—	0	Own data
1	0	IL8	—	0	Own data
1	0	—	AKAP12	0	21779438, Own data
1	0	—	PTEN	0	16246156

The last column lists the PubMed IDs of the respective publication.

Lastly, we note that our model still has limitations predicting the correct long-term behaviour for transiently activated genes such as *egr1*, *il8*, *ctgf* or *ccl20*. Although the model captures the long-term cell migration response and upstream pathway activity, it does not yet include a negative feedback down-regulating of these genes (compare Fig. 2A and B). Although the biological consequences of the transient gene activation remain unclear so far, this divergence between the model steady-state behaviour and experimental data beyond 3 h need to be addressed in more detail in the future.

**Fig. 3.**
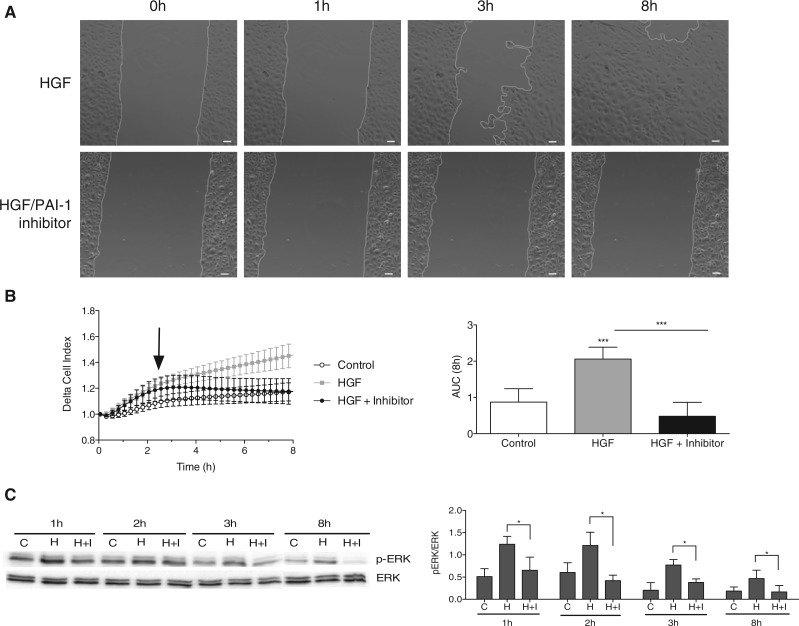
Keratinocyte migration analysis under HGF and PAI-1 inhibition. (**A**) Life-time imaging microscopy of NHK up to 8 h. HGF stimulation increases migration of NHK (upper panel), addition of PAI-1 inhibitor, Tiplaxtinin, decreases strongly the HGF effect (lower panel). Bar: 20 *μ*m. (**B**) Increased HGF migration is significantly reduced with PAI-1 inhibitor (25 *μ*M) addition after a short time period (left plot). Migration response is calculated as area under the curve (AUC; right plot). (C) Determination of pERK level under HGF and inhibition conditions. Original western blot of pERK and ERK for different time points reveals a continuous activation of pERK under HGF (H) stimulation over time comparing to control (**C**). In contrast under PA1-1 inhibition (H+I) the pERK level decrease significantly over time. * denote a *t*-test *P*-value < 0.05, data points obtained in triplicate. The ratio of pERK/ERK is shown for the respective time points (right plot)

Model simulation clearly reflects the necessity for the time sequential pathway activation, shown by the early and late steady state after 1 and 3 h, respectively. Initial HGF/MET receptor signalling triggers, while subsequent integrin/EGF receptor signalling sustains MAPK/ERK, PI3K, PLC/PKC and JNK/p38 signalling (Fig. 3C). Model simulations further predict that continued migration depends on both EGFR and integrin signalling. Failure of either one causes the down-regulation of the FAK protein and subsequent PI3K, MAPK/ERK and p38/JNK pathways (Fig. 2, Columns d and e). Indeed, we have previously shown the dependency of cell migration on sustained EGFR activity after MET signalling pathway activation (Busch *et al.*, 2008).

To experimentally validate the long-term dependency of NHK on the FAK protein-mediated integrin signalling, we interrupt the uPA/uPAR signalling pathway through inhibition of PAI-1. In line with previous findings (Providence and Higgins, 2004) and predicted from our network simulation, long-term, but not immediate cell migration should be decreased. A scratch assay (Fig. 3A) shows a significant decrease with the usage of the PAI-1 inhibitor (Tiplaxtinin) in migration when compared with control conditions. While under HGF stimulation, the scratch is almost closed after 8 h, hardly any cell movement is detected under additional PAI-1 inhibitor treatment. The delayed impact of PAI-1 inhibition becomes evident from a real-time analysis of keratinocyte migration using the xCELLigence Cell Analyzer (Fig. 3B). Clearly, the migration speed becomes strongly reduced after 2.5 h, in line with the suggested role for ‘late’ uPA/uPAR signalling pathway involvement, activating FAK through integrin/EGFR signalling for sustained migration. This time point also coincides with the start of transcriptional activation of PAI-1 (Fig. 2A), furthermore supporting the late involvement of this pathway. Interestingly, MET receptor and integrin/FAK pathways both activate MAPK/ERK, PI3K and PLC/PKC, RAC1/CDC42 and JNK/p38. However, while the former primes the cells towards migration, the impact of the latter on downstream pathways seems to be much more pronounced. Analysing the phosphorylation state of ERK (pERK) we observe an immediate and sustained increase of pERK under HGF treatment in line with our simulation. Differences in pERK level under simultaneous PAI-1 inhibition become most apparent at late time points beyond 2 h, confirming the model predictions of late impact of integrin signalling on ERK. Looking at FAK as an up-stream effector of ERK (Sawhney *et al.*, 2006), we find similar activity for pFAK under HGF stimulation and PAI-1 inhibition. We observe a strong, but late increase in FAK activity as determined from phosphorylation of the Y925 site of FAK (pFAK (Y925)). Simultaneous stimulation of HGF and PAI-1 inhibition decreases pFAK (Y925) when compared with HGF alone (Supplementary Fig. S1). Immediate increase of pFAK (Y925) suggests a possible role of FAK in the beginning of migration. Previous work has shown two distinct phases in FAK involvement for wound healing in rat keratinocytes (Providence and Higgins, 2004). There, the effect of PAI-1 inhibition became evident only after 6 h and more into migration. Herein, we can possibly explain the importance of FAK for prolonged migration through the time sequential regulation of uPA/uPAR, integrin and EGFR signalling pathways, which were predicted from network simulation and have been — in part — experimentally validated. Further studies for a better understanding of this complex pathway orchestration of HGF-induced migration will be necessary, of course.

## 4 CONCLUSIONS

We have shown for the first time a comprehensive model for HGF-induced keratinocyte migration. The model comprises two time scales and incorporates various signalling pathway critically involved in the initiation, sustaining and controlling of keratinocyte migration. Being mostly compiled from prior knowledge and vast literature, it will lend itself to rapid hypothesis testing of key points of interference.

We are aware that the above model cannot capture the entire complexity of this process. Most of the simulation results are suggestive about the underlying process and further experiments will need to be conducted to study the complex orchestration of pathways leading to keratinocyte migration. However, our model makes several important and experimentally testable predictions about putative targets and time of intervention to control this process. It underscores the temporal sequence of events from initial trigger to execution, allowing for a fail-safe mechanism of migration under wild-type and inhibition conditions. It is in line with previous findings from literature and captures the short- and long-term feedback regulation of protein signalling and gene expression. In particular, the order of events becomes evident, how MET receptor activity primes the cell for subsequent EGFR and integrin signalling, leading sustained migration. Exchanging the order of stimulation (first EGFR followed by MET receptor activation), keratinocytes will not migrate, which is explained in the model and supported by our first experimental results from the altered role of the FAK proteins at late time points. Indeed, MET receptor deregulation is a hallmark for cancer metastasis (Gherardi *et al.*, 2012). If MET is constitutively activated, it would need only additional EGFR activity to induce cellular spread, according to the network model. Apparently, this by-passes the fail-safe mechanism for cells to migrate only within the correct context. A synergistic action of EGFR and MET have been found before (Brusevold *et al.*, 2012; Zhou *et al.*, 2007), yet the causal relationship between them remained unclear so far.

Beyond further experimental validation, the need for model extensions to multiple logical states or even continuous representation of the variables using ordinary differential equation approach is evident. Our current approach cannot account for the multiple negative feedback regulations of either AKAP12 or DUSP1. Such negative feedback seems to be a recurrent regulatory motif (Becskei and Serrano, 2000; Blüthgen, 2010), and it should be interesting to study its biological implications.

Taken together, the Boolean model approach lends itself to a better mechanistic understanding of the process of keratinocyte migration, wound healing, cancer metastasis and/or impaired wound healing from stimulation to phenotype development and will help in the prediction of cellular control targets in wild type and disease.
